# [Corrigendum] Arctigenin inhibits triple-negative breast cancers by targeting CIP2A to reactivate protein phosphatase 2A

**DOI:** 10.3892/or.2025.8925

**Published:** 2025-06-05

**Authors:** Qiuyue Huang, Shanshan Qin, Xiaoning Yuan, Liang Zhang, Juanli Ji, Xuewen Liu, Wenjing Ma, Yunfei Zhang, Pengfei Liu, Zhiting Sun, Jingxuan Zhang, Ying Liu

Oncol Rep 38: 598-606, 2017; DOI: 10.3892/or.2017.5667

Following the publication of the above article, the authors drew to the Editor's attention that they has misclassified some of their original data, and this led to the erroneous compilation of the cell invasion and scratch wound assay data shown in Fig. 5A and B respectively on p. 604. Moreover, the authors realized that the same GAPDH control western blotting data had inadvertently been included in Fig. 4A and G on p. 603, where these data were correctly shown only for Fig. 4G.

However, the authors had retained their original data for these figures, and the revised versions of Fig. 4 and Fig. 5, now showing the correct data for the GAPDH bands in Fig. 4A and the correct data for Fig. 5A and B, are shown on the next two pages. Note that the errors made in terms of the assembly of the data in these figures did not affect the overall conclusions reported in the paper. The authors are grateful to the Editor of *Oncology Reports* for granting them this opportunity to publish a Corrigendum, and apologize to both the Editor and the readership for any inconvenience caused.

## Figures and Tables

**Figure 4.**
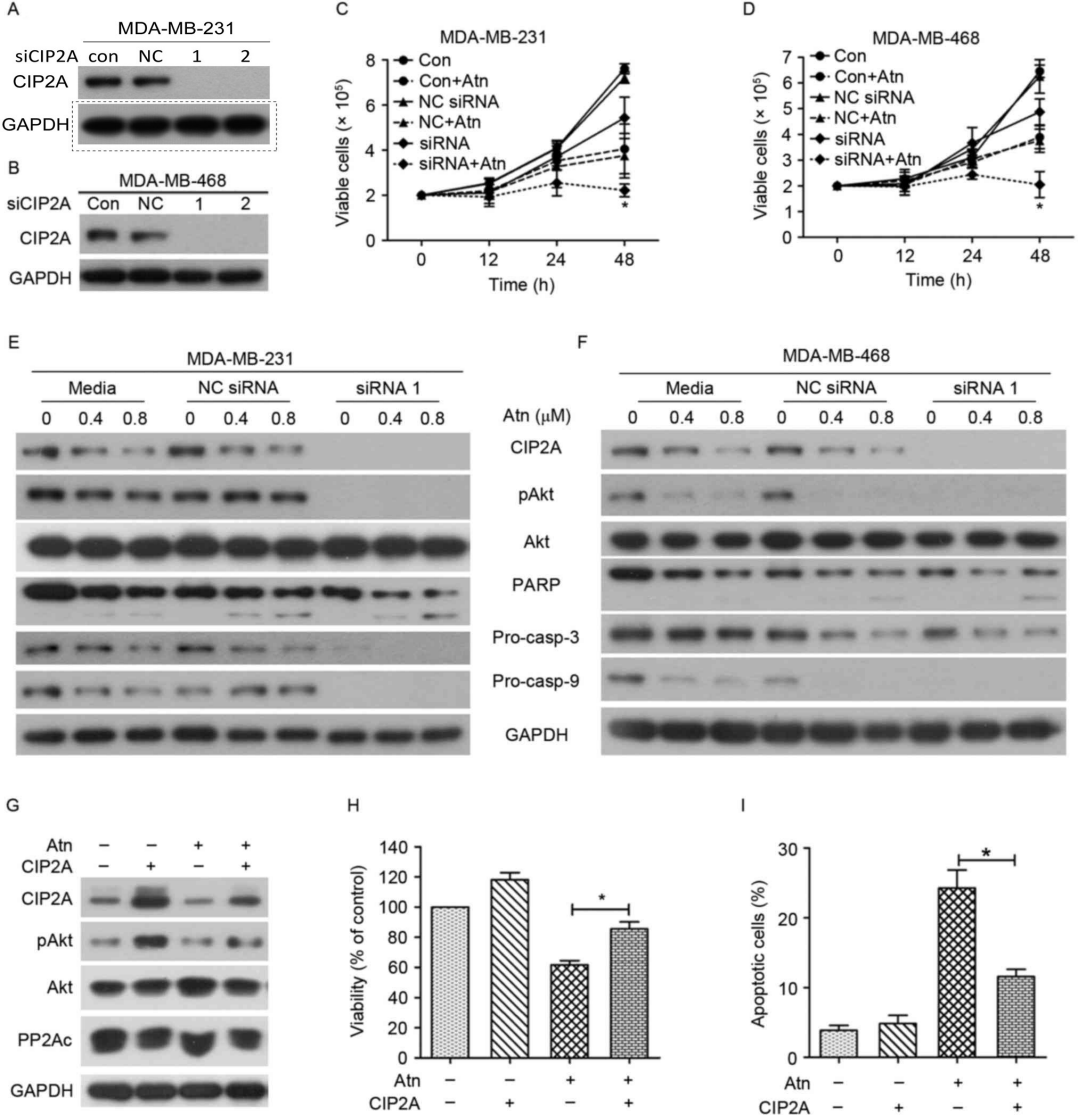
Targeting CIP2A/PP2A/pAkt pathway promotes molecular mechanism of Atn-induced TNBC cell apoptosis. (A and B) MDA-MB-231 and MDA-MB-468 cells were transfected with 100 nM CIP2A-specific siRNA or NC siRNA for 48 h. Cells were harvested for western blot analyses. (C and D) MDA-MB-231 and MDA-MB-468 cells were transfected with CIP2A-specific siRNA or NC siRNA, followed by treatment with Atn (0.4 µM) for indicated times. (E and F) MDA-MB-231 and MDA-MB-468 cells were transfected with CIP2A-specific siRNA or NC siRNA, followed by treatment with Atn (0, 0.8 and 1.2 µM) for 24 h. Cells were harvested for western blot analyses. (G-I) MDA-MB-231 cells were transfected with a CIP2A expression plasmid, and then western blotting, MTT, and flow cytometry were used to detect protein expression, proliferation, and apoptosis 48 h after transfection.

**Figure 5.**
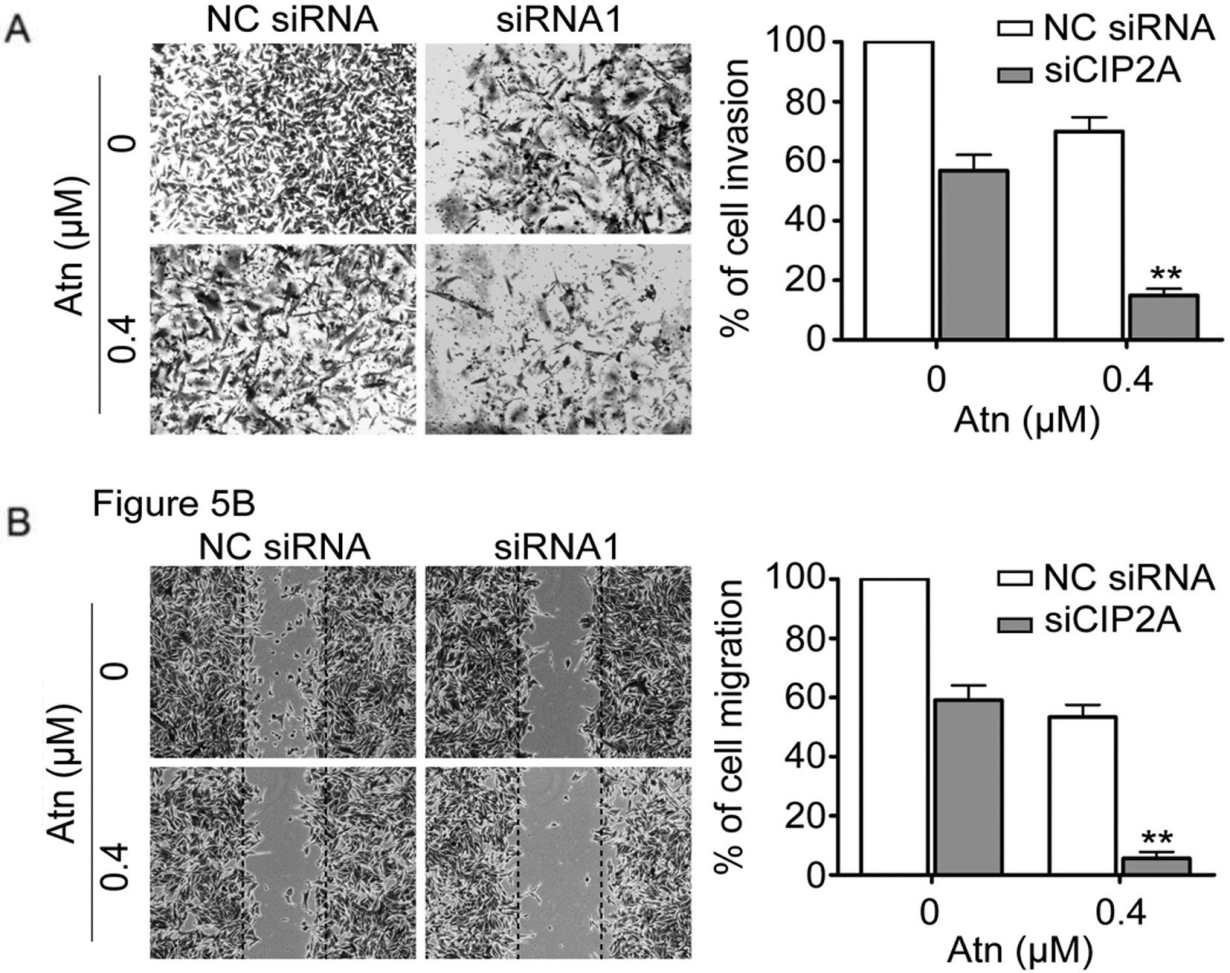
Silencing CIP2A enhances Atn-inhibited invasive behavior of TNBC cells. (A) MDA-MB-231 cells were transfected with 100 nM CIP2A-specific siRNA or NC siRNA, followed by treatment with Atn (0.4 µM) for 24 h. To evaluate cell invasion, the cells were analyzed for 20 h by invasion assay. (B) MDA-MB-231 cells were transfected with 100 nM CIP2A-specific siRNA or NC siRNA, followed by treatment with Atn (0.4 µM) for 24 h. To evaluate cell migration, the cells were analyzed for 24 h by wound healing assay. **P<0.01 vs. NC siRNA.

